# Artemisinins induce endoplasmic reticulum stress in acute leukaemia cells in vitro and in vivo

**DOI:** 10.1002/jha2.314

**Published:** 2021-10-16

**Authors:** Rubia Isler Mancuso, Juliana Hofstätter Azambuja, Fernanda Soares Niemann, Ada Congrains, Mary Ann Foglio, Eduardo Magalhães Rego, Sara Teresinha Olalla Saad

**Affiliations:** ^1^ Haematology and Transfusion Medicine Center University of Campinas Campinas Brazil; ^2^ Faculty of Pharmaceutical Science University of Campinas Campinas Brazil; ^3^ Center for Cell Based Therapy University of São Paulo Ribeirão Preto Brazil; ^4^ Haematology Division, LIM31, Faculdade de Medicina University of São Paulo São Paulo Brazil

**Keywords:** acute leukaemia, artemisinin, artesunate, endoplasmic reticulum stress

## Abstract

Loss of endoplasmic reticulum (ER) homeostasis leads to ER stress, thus prolonged activation can lead to apoptosis. Herein, artesunate (ART) induced ER stress in leukaemia cells, resulting in eIF2α phosphorylation, activation of transcription factor 4, subsequent CHOP upregulation and XBP1 splicing. Furthermore, in vitro cyclin/CDKs reduction induced G1‐phase arrest. An in vivo xenograft model showed a consistent pattern of ART in reducing tumour burden, supporting roles in the UPR pathway, which we speculate could lead to apoptosis by NOXA activation. Moreover, ART were capable of increasing the survival of mice. Taken together, our data indicate that ART may represent an interesting weapon to fight leukaemia.

Acute myeloid leukaemia is a malignant clonal expansion of progenitor cells in the bone marrow, characterized by the differentiation arrest of myeloid progenitor cells [1]. Curative therapies, including intensive chemotherapy and allogeneic hematopoietic stem cell transplantation, are generally applicable to a minority of younger patients, whereas most elderly individuals exhibit poor prognosis and survival [2]. Thus, new therapeutic approaches with low toxicity and high potency are required.

Artemisinin (ARS), a natural compound that has been used for more than two millennia in traditional Chinese medicine as a remedy for fevers and chills, is currently used to treat malaria with no side effects [3]. ARS derivatives are sesquiterpene lactones with an endoperoxide moiety that is essential for activity [4]. In addition to the range of beneficial effects, ARS has shown anticancer activities in vitro and in vivo, including apoptosis induction, cell cycle arrest and oxidative stress response [4, 5].

Herein, we report our in vitro and in vivo results regarding the use of two ARS derivatives in leukaemia, artesunate (ART) and artemether (ARM), which are water and oil soluble, respectively. The endoplasmic reticulum (ER) represents a complex membranous network that mediates the folding and trafficking of transmembrane and secretory proteins [6]. The unfolded protein response (UPR) pathway consists of three ER transmembrane proteins, including inositol‐requiring protein‐1 (IRE1), PKR‐like ER kinase (PERK) and activating transcription factor 6 (ATF6). Loss of ER homeostasis leads to ER stress, which can be induced by various pathophysiological insults, including oxidative stress [7]. Severe or prolonged ER stress and uncontrollable UPR can activate ER stress‐associated cell death signalling [8]. Therefore, activation of ER stress could represent a strategy to lead cancer cells to death and control cancer progression [9].

In our study, we detected decreased proliferation of the leukaemia cell lines, U937 (IC_50_ of 3.59 μM) (Figure 1A) and HL‐60 (IC_50_ of 0.35 μM) (Figure S1A), at 24 h after treatment, dependent on the concentration and time of ART treatment. Additionally, ARM also reduced the viability of both cell lines (IC_50_ of 17.1 and 26.7 μM after 24 h of ARM treatment, in U937 and HL‐60, respectively) by MTT assay (Figure S1B,C). No toxicity was observed in peripheral blood mononuclear cells (PBMCs), suggesting a selective anti‐tumour effect (Figure S1D,E). Of note, at 24 h of ART treatment, apoptotic cells were observed in U937 (Figure 1B) and HL‐60 cultures (Figure S1F). ART presented a higher potential to kill leukemic cells, compared to ARM (Figure S1G,H). Our data showed a strong NOXA expression in U937 cells after 24 h of ART treatment (Figure 1C). The increase of NOXA expression has been shown by other [10]. Therefore, our data corroborate that of other ARS derivatives.

**FIGURE 1:**
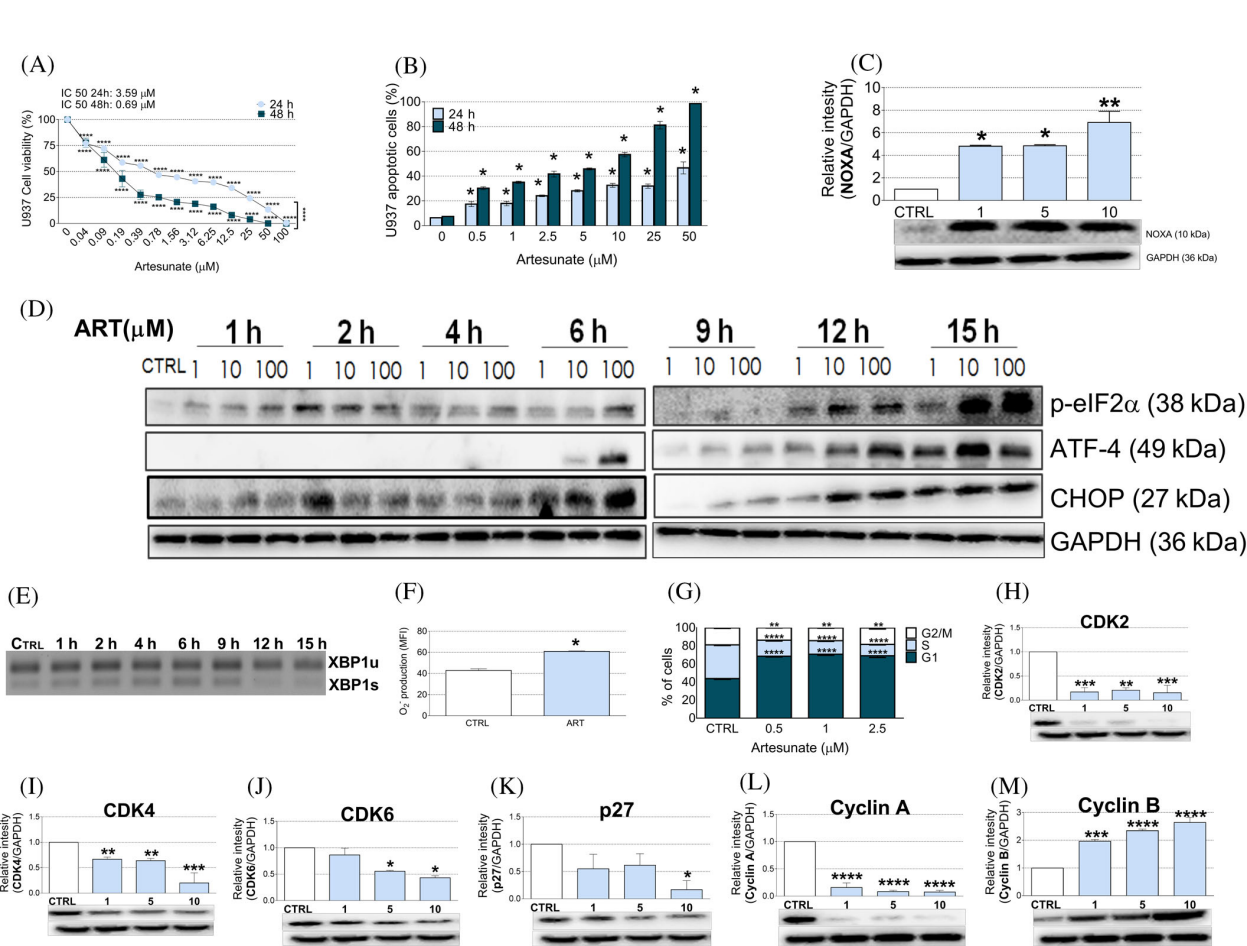
ART derivatives induce apoptosis mediated by NOXA and activation of the ER stress pathway in leukemic cells. (**A**) U937 leukemic cells were exposed to increasing concentrations of ART, and cell viability was assessed by MTT following 24 and 48 h of treatment. (B) U937 cells were exposed to increasing concentrations of ART. Cell apoptosis was assessed by Annexin V and flow cytometry. (C) ART changes in NOXA protein after 24 h and (D) the expression of p‐eIF2α, ATF4 and CHOP were analysed in U937 cells treated with ART at different time points. GAPDH was used as loading controls. Relative luminescence units (RLU), compared to untreated cells, are shown (mean ± SEM, *n* = 3 [cell lines]). (E) XBP1 mRNA was analysed by standard RT‐PCR after ART treatment of U937 cells at different time points. The upper band represents 210 bp (unspliced) and the lower band represents 184 bp (spliced). (F) U937 cells were exposed to IC_50_ of ART. FACS was used for determination of O_2_^–^ production after 18 h. (G) U937 cells were exposed to increasing concentrations of ART for 24 h. FACS was used for determination of cell cycle distribution. (G–M) ART changes the expression of cell cycle‐related proteins after 24 h. Control cells were exposed to DMSO. Data were analysed by ANOVA, followed by post hoc comparisons (Tukey–Kramer test). *p < 0.05, **p < 0.01, ***p < 0.001 and ****p < 0.0001, significantly different from control cells

Interestingly, our data showed an activation of the PERK and IRE1 branches in vitro, which increase proteins, such as p‐eIF2α, ATF4, CHOP, and XBP1 splicing. An increase in phosphorylation of α‐subunit of the eukaryotic translation initiation factor‐2 (p‐eIF2α), which is believed to sense accumulating misfolded protein and attenuation of global translation, was detected transiently after ART treatment, followed by an increase in the transcription factor 4 (ATF4), 6 h later. ATF4 has the ability to target CHOP and switch to a terminal outcome in cases where ER stress response is not resolved [11]. Studies indicated NOXA as an important activator of apoptosis in response to ER stress [12]. Finally, an increase in CHOP protein expression was observed at 6 h after ART treatment, which was maintained until 15 h in U937 (Figure 1D) and HL‐60 cells (Figure S1J). In addition, we observed an increase of XBP1 splicing in U937 (Figure 1E) and HL‐60 (Figure S1I) cultures after ART treatment with a continuous increase until 9 h followed by a decrease. We further observed an enhanced in sXBP1 protein expression in the HL‐60 cell line after 6 h followed by another increase after 12 and 15 h after ART treatment (Figure S1J). Thus, our data corroborate the results recently published by Moses and colleagues [13] and suggest that the effects of ART on leukaemia cell lines are mediated, at least in part, by activation of ER stress, thereby reaffirming that CHOP levels may serve as a biomarker for artemisinin actions.

Multiple disturbances can cause accumulation of unfolded proteins in the ER, such as redox regulation induced by oxidants, leading to protein unfolding and misfolding [8]. We measured superoxide levels in the cytosol of U937 cell lines by Mitosox (Thermo Fisher, Waltham, MA, USA) following 18 h of ART treatment (3.5 μM) and found an increase in O_2_^–^ production (Mean Fluorescence Intensity (MFI) from 42.78 ± 3 to 60.87 ± 0.90) (Figure 1F). Our data further showed a G1‐phase arrest at 24 h after ART treatment of U937 cells (43.75% to 68.9% with 2.5 μM; *p* < 0.0001) (Figure 1G), contributing to a decrease of cyclin A (10‐fold) (Figure 1L) which is repressed during the G1‐phase of the cell cycle and activated at the S‐phase entry. In addition, a decrease in the expressions of CDK2 (5‐fold) (Figure 1H), CDK4 (5‐fold) (Figure 1I) and CDK6 (2‐fold) (Figure 1J), with no alterations in p27 expression with low doses (Figure 1K), were observed. Furthermore, ART induced an increase in cyclin B (Figure 1M), expressed during the G2/M‐phase of cell cycle.

Additionally, in our xenograft model (Figure 2A), ART treatment (200 mg/kg/i.p.) reduced tumour growth (Figure 2B). Our data demonstrated a reduction in tumour volume (from 1461 ± 178 to 702 ± 216 mm^3^, *p* = 0.0071) (Figure 2C) and in tumour mass at the end point (from 1.37 ± 0.12 to 0.73 ± 0.15 g, *p* = 0.0044) in ART‐treated mice, compared with mice treated with 5% sodium bicarbonate in saline solution (Figure 2D). No differences were observed in mice weight (Figure 2E). Additionally, an increase in CHOP (*p* = 0.0012) (Figure 2F) and a decrease in CDK2 (*p* = 0.039) (Figure 2G) protein expression were observed in ART‐treated mice. Interestingly, despite not having observed any significant difference, three mice (out of five) showed increased NOXA expressions (*p* = 0.209) (Figure 2H), p‐eIF2α (*p* = 0.121) (Figure 2I), p21 (*p* = 0.126) (Figure 2J) and p27 (*p* = 0.0687) (Figure 2K). No difference was observed in cyclin A (*p* = 0.211) (Figure 2L) and CDK4 (*p* = 0.716) (Figure 2M). Thus, in our in vivo results we show, to our knowledge for the first time, the activation of the PERK branch in leukemic cells when treated with ART.

**FIGURE 2:**
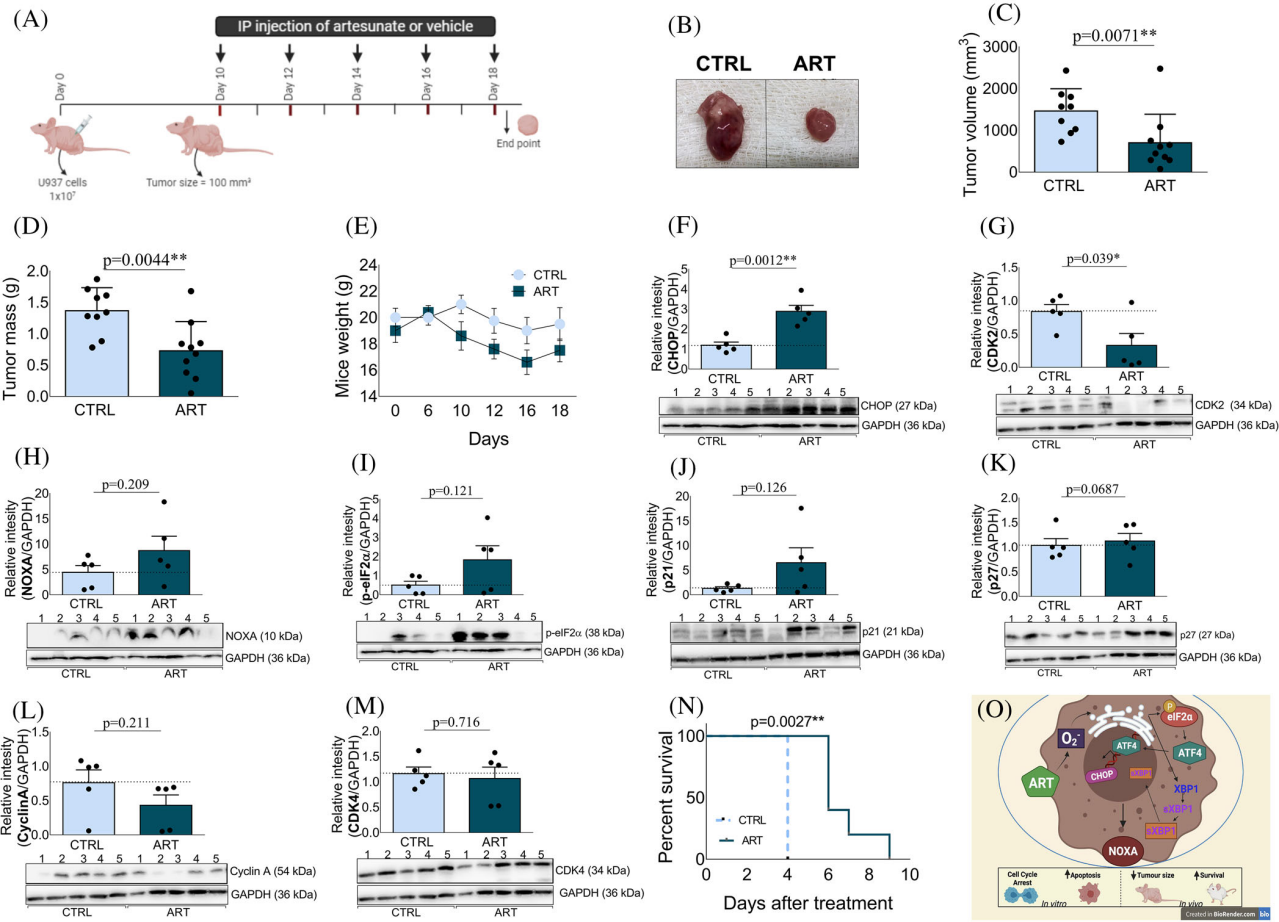
Effects of ART upon the activation of the PERK branch of the ER stress pathway of U937 xenograft tumours and upon the survival of the PML–RARa model. (A) Schematic diagram showing transplantation of 1 × 10^7^ U937 cells into NOD/SCID mice (Ethics Committee Number 4957‐1/2018) (day 0), followed by control or ART (200 mg/kg) i.p. injections on days 10, 12, 14, 16 and 18. (B) Representative images of tumours after ART treatment for 18 days (compared to controls), showing tumour size reduction. (C) The representative column diagrams show tumour volume weight and (D) tumour mass. The changes in mice weight (E) over the course of time of the experiment in the U937 xenograft model are shown. Western blot analyses of (F) CHOP, (G) CDK2, (H) NOXA, (I) p‐eIF2α, (J) p21, (K) p27, (L) cyclin A and (M) CDK4. (N) ART treatment significantly prolonged survival of the PML–RARa model, showing that ART has antileukemic activity in vivo. (O) Illustration of artesunate hypothetic mechanism in leukaemia cells. The bar graphs show means ± SEM of relative luminescence units (RLU), compared to vehicle mice (*n* = 5). GAPDH was used as the loading control. **p < 0.01, ***p < 0.001 and ***p < 0.0001, significantly different from control groups

Finally, a survival analysis of the acute promyelocytic leukaemia model [14, 15] treated with ART was conducted. For the generation of this model, NOD/SCID mice were sub‐lethally irradiated with 2 Gy, and 1 × 10^6^ leukemic cells were intravenously injected into the tail vein [16]. Blood counts were monitored weekly and, after the confirmation of leukaemia (12th day), mice (*n* = 10) were submitted to daily intraperitoneally injections of ART (25 mg/kg, i.p.) until death. An extended survival of ART‐treated mice (*p* = 0.0027) was observed, compared to untreated mice (Figure 2N).

In summary, our data demonstrate a consistent pattern of ART in reducing tumour burden in the xenograft model, supporting roles in UPR pathway, principally the PERK branch. We speculated that the CHOP‐target gene NOXA regulated the cell fate. Moreover, ART was capable of increasing the survival of the PML–RARa model. ART reduced leukaemia cell growth, accompanied by increased apoptosis, G1‐phase cell cycle arrest and the reduction of CDKs and cyclin A in vitro. Herein, we hypothesized an important direct antileukemic potential for ART‐type drugs (Figure 2O). Recently, we demonstrated an indirect antileukemic effect of ART through the modulation of monocytes to a tumoricidal phenotype [17]. Taken together, our data indicate that ART may represent an interesting weapon to fight leukaemia.

## AUTHOR CONTRIBUTIONS

R. I. Mancuso conceptualized the study, performed the experimental work, data analysis, and wrote the original draft. J. H. Azambuja performed data analysis, reviewed and edited the manuscript. F. S. Niemann carried out the experimental work and data analysis. A. Congrains performed data analysis, reviewed and edited the manuscript. M. A. Foglio and E. M. Rego contributed with essential reagents and reviewed the manuscript. S. T. Olalla Saad acquired funding, supervised the study, wrote and reviewed the manuscript.

## CONFLICT OF INTEREST

The authors declare no conflict of interest.

## Supporting information



Supplementary Fig 1. ARS derivatives reduce cell proliferation and induce apoptosis in leukemic cells. (A‐E) HL‐60 and U937 leukemic cells and PBMCs were exposed to increasing concentrations of ART and ARM. Cell viability was assessed by MTT following 24 and 48h of treatment. (F‐H) HL‐60 and U937 leukemic cells were exposed to increasing concentrations of ART and ARM. Cell apoptosis was assessed by Annexin V and flow cytometry. (I) XBP1 mRNA were analysed by standard RT‐PCR after ART treatment of HL‐60 cells at different time points. The upper band represents 210 bp (unspliced) and the lower band represents 184 bp (spliced). (J) the expression of p‐eIF2α, ATF‐4, CHOP, and XBP1s were analysed in HL‐60 cells treated with ART at different time points. GAPDH was used as loading controls. Relative luminescence units (RLU), compared to untreated cells are shown (mean ± SEM, n = 3 [cell lines]). Data were analysed by ANOVA, followed by post hoc comparisons (Tukey‐Kramer test). *p  <  0.05, **p  <  0.01, ***p  <  0.001 and ****pspan style = “font‐family:Arial; background‐color:#fcfcfc” >   <  0.0001, significantly different from control cells.
